# Molecular dynamics simulations and experimental studies reveal differential permeability of withaferin-A and withanone across the model cell membrane

**DOI:** 10.1038/s41598-021-81729-z

**Published:** 2021-01-27

**Authors:** Renu Wadhwa, Neetu Singh Yadav, Shashank P. Katiyar, Tomoko Yaguchi, Chohee Lee, Hyomin Ahn, Chae-Ok Yun, Sunil C. Kaul, Durai Sundar

**Affiliations:** 1 AIST-INDIA DAILAB, DBT-AIST International Center for Translational and Environmental Research (DAICENTER), National Institute of Advanced Industrial Science and Technology (AIST), Tsukuba, 305 8565 Japan; 2 DAILAB, Department of Biochemical Engineering and Biotechnology, Indian Institute of Technology (IIT) Delhi, Hauz Khas, New Delhi, 110 016 India; 3 Department of Bioengineering, College of Engineering, Hanyang University, 222 Wangsinmi-ro, Seongdong-gu, Seoul, 04763 Republic of Korea; 4 GeneMedicine Co., Ltd, 222 Wangsimni-ro, Seongdong-gu, Seoul, 04763 Republic of Korea; 5 Institute of Nano Science and Technology (INST), 222 Wangsimni-ro, Seongdong-gu, Seoul, 04763 Republic of Korea

**Keywords:** Biological techniques, Biophysics, Computational biology and bioinformatics

## Abstract

Poor bioavailability due to the inability to cross the cell membrane is one of the major reasons for the failure of a drug in clinical trials. We have used molecular dynamics simulations to predict the membrane permeability of natural drugs—withanolides (withaferin-A and withanone) that have similar structures but remarkably differ in their cytotoxicity. We found that whereas withaferin-A, could proficiently transverse through the model membrane, withanone showed weak permeability. The free energy profiles for the interaction of withanolides with the model bilayer membrane revealed that whereas the polar head group of the membrane caused high resistance for the passage of withanone, the interior of the membrane behaves similarly for both withanolides. The solvation analysis further revealed that the high solvation of terminal O5 oxygen of withaferin-A was the major driving force for its high permeability; it interacted with the phosphate group of the membrane that led to its smooth passage across the bilayer. The computational predictions were tested by raising and recruiting unique antibodies that react to withaferin-A and withanone. The time-lapsed analyses of control and treated cells demonstrated higher permeation of withaferin-A as compared to withanone. The concurrence between the computation and experimental results thus re-emphasised the use of computational methods for predicting permeability and hence bioavailability of natural drug compounds in the drug development process.

## Introduction

Drug design and development is a multidimensional and extensive regimen. Many potent drugs fail in later stages of trials leading to huge loss of time and resources. Inappropriate pharmacokinetics of molecules is one of the major causes of such failure. These pharmacokinetic properties generally include low bioavailability, short elimination half-life and variable behavior due to genetic or environmental factors. Especially for orally administered drugs, bioavailability is very critical for proper absorption that largely depends on their solubility and membrane permeability.

In eukaryotic systems, both active and passive transport modes are available for the transport of a molecule through a lipid membrane^1^. Active transport requires ATP to transport the molecule across a membrane, while passive transport involves diffusion of molecule across the membrane without any external assistance or energy input. However, the rate of passive diffusion across a bilayer is proportional to the partition coefficient between membrane and external medium, diffusion coefficient of the compound through the membrane, and concentration gradient of compounds across the bilayer. Further, the important physico-chemical properties responsible for the process of membrane binding and diffusion are lipophilicity, molecular weight and polarity^2^.

Studies on characterization and kinetics of drug permeability have shown that majority of molecules get absorbed in the body via passive diffusion for which a drug must penetrate the apical membrane^3,4^. Thus, passive transport across the apical cell membrane represents an essential step of drug design, bioavailability and efficacy. Membrane penetration potential analysis of candidate drugs becomes important for elimination of molecules that may fail later due to poor bioavailability or chose the ones with specificity for only the diseased cells.

**Figure 1:**
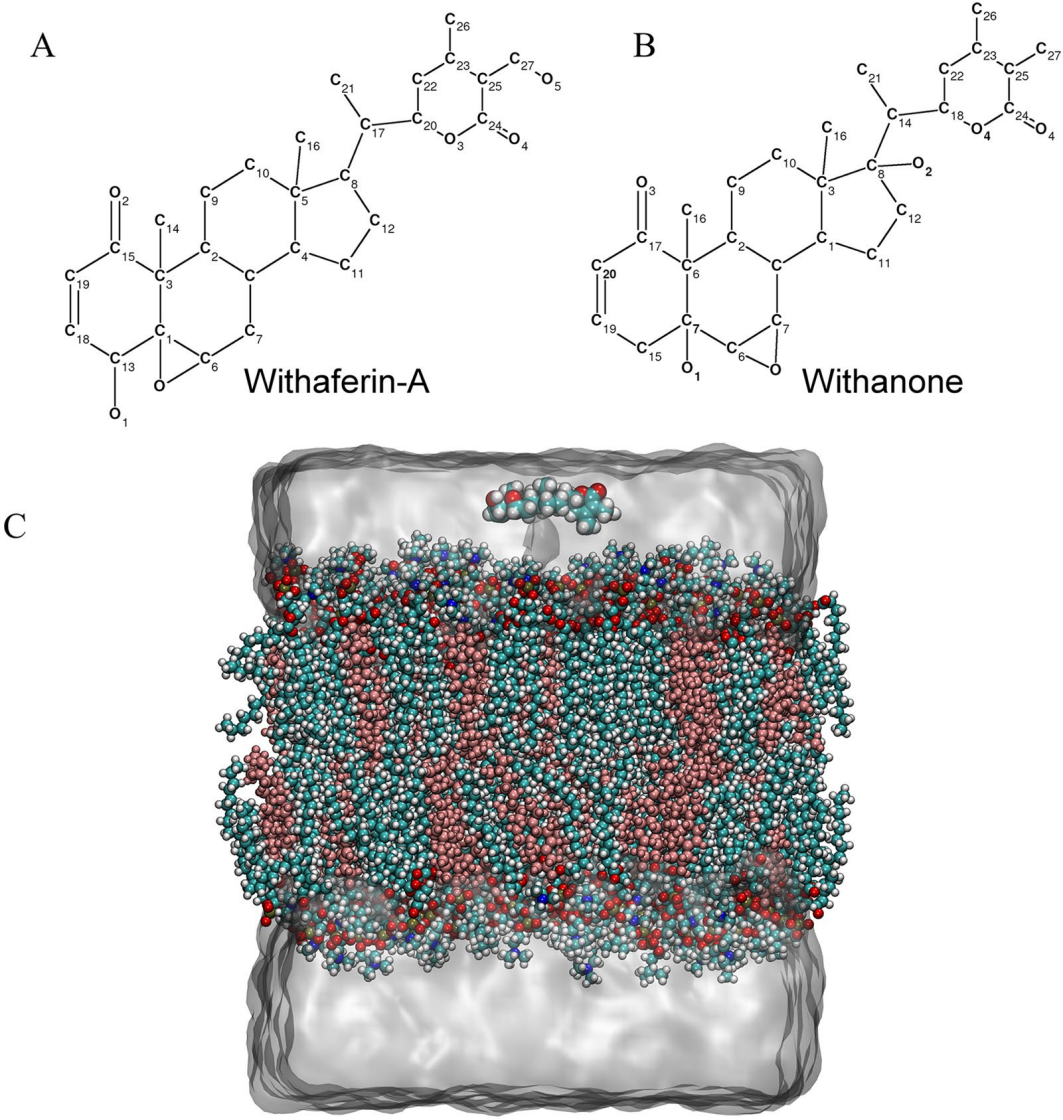
The 2D pictorial representation of the natural compounds: (**A**) withaferin-A and (**B**) withanone. (**C**) The representation of the system used in MD simulations: a drug molecule (in vdw representation at the top of membrane) interacts with and traverse through a bilayer surrounded by water.

Withanolides (C_28_-steroidal lactones), a class of secondary metabolites from Solanaceae plant family are long known to possess anti-cancer^5–10^, anti-stress, anti-neurodegenerative^11^, and anti-microbial activities^12^. Withaferin-A extracted from Ashwagandha (*Withania somnifera*) has been studied the most amongst all withanolides that share the same core structure^13^. Interestingly, despite having quite similar structures, Withaferin-A (Wi-A) and Withanone (Wi-N) differ remarkably in their activities. Wi-A is a C1, C6 epoxy compound carrying hydroxyl groups on C13 and C27, while Wi-N is a C6, C7 epoxy compound having hydroxyl groups on C7 and C8 (Fig. 1). Wi-N, although less potent as compared to Wi-A in cytotoxic assays for cancer cells, has been earlier shown to be safe for normal cells^14–17^. Various factors such as molecular size, structural conformations, type and position of the functional groups affect the interactions of withanolides with their biological targets. Permeability across cell membrane can also be anticipated to be the reason behind this differential activity.

Though experimental^18–20^ and theoretical studies^21–23^ have been carried out in the past for investigating drug permeability, Molecular Dynamics Simulations (MDS) is now widely used for the estimation of partition of a drug in a membrane^24,25^. Since in vivo studies of intestinal membrane permeability are expensive and may prove potentially harmful for the volunteers, the in vitro models such as partitioning in isotropic system^26,27^, transport across artificial membranes^28^ and transport across cultured epithelial cell monolayer^29,30^ have been the valuable tools. However, these models do not take into account the molecular properties of membrane that play an important role in governing the permeability of a solute. Computational methods, on the other hand, hold remarkable potential to investigate the structure-permeability relationships. Nonetheless, full atom computational models of membranes such as POPC (1-palmitoyl-2-oleoyl-sn-glycero-3-phosphocholine), POPS (1-palmitoyl, 2-oleoyl-sn-glycero-3-phosphoserine) etc. have been used to study the interactions of small molecules with the membrane and their permeability characteristics. These membrane models exhibit the structural and dynamic features of membrane bilayer, which have been investigated and verified by MDS^31,32^.

Cholesterol is one of the major components of eukaryotic cell membrane and also reported to have a large effect on the membrane permeability^33^. Generally solute permeability across the high cholesterol content membranes is low^34–36^. By measuring the potential of mean force (PMF) barriers of dynamic drugs, hypericin and its derivatives, it has been reported that cholesterol significantly influences the permeation of these molecules^37^. It was shown that the calculated rate of permeation is lower at high cholesterol content due to increase in PMF barrier. Therefore, adding cholesterol molecules to POPC lipid membrane helps in bringing the simulation environment closer to the actual cell membrane system. Such mixed membrane models have been used successfully to predict the permeability of drugs such as ibuprofen, cimitedine, six-β-blocker, aliphatic amine, and carboxylic acid drugs using the MDS^38,39^.

The partition of a drug could be either predicted by long nano-second classical MDS^40,41^, or by the Potential of Mean Force (PMF) methods^42,43^. Using MDS on various fluorescent azaaromatic probes on water-membrane, it has been earlier shown that the probe localization is determined by the electrostatic dipole–dipole and van der Waals interactions^44^. Further, it was also shown that 2,6 Bis (1H-Benzimidazole-2-yl) pyridine (BBP) preferred to locate around 15-16 Å from the DPPC (1,2dipalmityol-sn-glycero-3-phosphatidycholine) membrane center^42^. A MDS study of 17 amino acids in lipid bilayer showed that the partitioning of charged and polar side chain amino acids were accompanied by water defects^45^. Furthermore, the solute size, polarizability and free energy of transfer from water to membrane also affected the transfer of solute from water into membrane.

In the present study, we have elucidated the mechanism of permeation of two natural drug molecules (Wi-A and Wi-N) through POPC bilayer using computer simulations, which were further validated by recruiting unique withanolide-recognizing antibodies in cell-based assays. In-spite of having very little difference in their structures, Wi-A molecules successfully crossed the bilayer with low free energy associated with it, while the passage of Wi-N was associated with high free energy barrier. We present here a computational assay which would be useful for predicting permeation and bioavailability of drug compounds in drug development process.

## Methods

### Parameterization and placement of withanolides

The geometry optimization and the charge of Wi-A and Wi-N molecules were calculated at 6-31G* level by using GAMESS-US^40^ and further refinement was carried out by using Restrained Electrostatic Potential Fit (RESP) using the RED-Tool package^41^. In each system, one drug molecule was placed in the aqueous phase by aligning the the plane of the molecule with the plane of the membrane (Fig. 1).

### Molecular dynamics (MD) simulations

Three MD simulations- (1) POPC membrane, (2) POPC membrane having Wi-A, and (3) POPC membrane with Wi-N, were carried out. The model membrane was composed of POPC and cholesterol molecules, where, the starting structures were taken from a 2 μs long equilibration simulation data^46^. The bilayer membrane model consisted of 70 POPC and 35 randomly placed cholesterol molecules per layer or leaflet. All simulations were run using TIP3P water models^47^ and amber Lipid14 force field parameter^48^. It has been earlier shown that Lipid14 is a good choice for molecule permeability calculations^49^, all the structures were converted to the Lipid14 naming convention by using charmmlipid2amber.py script, available with Amber Tools v18. The prepared models were then completely solvated using 7499 TIP3P water molecules. To attain the charge neutrality, adequate numbers of Na^+^ and Cl^–^ ions were added in each simulation box.

### Equilibration and production procedure

The solvated membrane in the simulation box was then equilibrated using a slightly modified version of the multi-step protocol^50^. Briefly the procedure consisted of altering cycles of steepest descent (1000 steps) and conjugate gradient energy minimization (1000 steps) followed by position restrained MD with strong harmonic restraints on all non-hydrogen atoms of the bilayer membrane and solvent. In successive steps, the positional restraints were gradually relaxed. The detailed description of the minimization and equilibration protocol can be found in the supplementary material S1. All the energy minimization and MD simulations were run using the SANDER modules of the AMBER18 program suite^51^. All the three systems (Control, POPC-Wi-A, and, POPC-Wi-N) were equilibrated using a slightly modified version of the multi-step protocol The production simulations for all the systems were run for 600 ns in NPT ensemble using GPU accelerated version of the PMEMD program of AMBER simulation suite^52,53^. The temperature and pressure of the systems were kept restrained to 310 K above the crystalline fluid/liquid phase transition temperature and 1 atm respectively using the Langevin thermostate, while the aniosotrophic Berendsen barostat^50^ was used for pressure control. All bonds involving hydrogen atoms were constrained using the SHAKE algorithm. Short range electrostatics were calculated up to 10 Å and the Particle Mesh Ewald^51^ algorithm was used to calculate the long-range electrostatics. An integration time step of 2 fs was used, and structures were saved every picosecond resulting in around 6,00,000 structures. Since the transition of withanolides from bulk phase to lipid starts at ~ 33 ns, the analysis was performed on the last 570 ns of the datausing the CPPTRAJ module of Amber suite.

### Potential of mean force (PMF) simulations

The potential of mean force (PMF) free energy profiles for the partitioning of drug compounds were calculated using umbrella-sampling simulations. The withanolides were placed on the water phase on top of the membrane. Using a pulling rate of 1 Å/ns, the drugs were then pulled out from the aqueous phase towards the center of bilayer membrane system for a total of 38 Å (force constant of 1.5 kcal/mol/Å^2^) in the NPT ensemble with semi-isotropic pressure coupling scheme. During the simulations, snapshots were saved every 1 Å from the top (z = 38) of membrane to center (z = 0) generating 38 windows. The results were calculated for one bilayer, and due the symmetry of membrane it was assumed that the other half behaves similarly. This was achieved by reflecting the data along z axis and adding 37 or 38 windows depending on whether or not the window at z = 0 Å was reflected as well. Each umbrella sampling window was run for 30 ns of production run with the force constant of 3.0 kcal/mol/Å^2^, totaling ~ 1 μs of sampling per permeant. Configurations were saved every 1 ps. Further, to calculate the PMF, the biased distributions were reweighted using Weighted Histogram Model (WHAM)^54,55^.

### Diffusion and resistance calculations

Theoretically, the permeability coefficient of a molecule can be computed from an atomistic simulation-based PMF approach via the inhomogeneous solubility diffusion permeability model^56^. Here, the permeation is divided into a three-step process: (a) involving the partitioning of a permeant from the aqueous phase on the side of leaflet, (b) diffusion across the bilayer and (c) partitioning from the other side of bilayer into aqueous phase.

$$ \frac{1}{P} = R = \mathop \int \limits_{{z_{1} }}^{{z_{2} }} \frac{{e^{{\beta \left[ {w\left( z \right)} \right]}} }}{D\left( z \right)}dz $$

where, $R\left( z \right)$ is the resistivity of every “slice” of the membrane at position “z”. $w\left( z \right)$ is the PMF, $D\left( z \right)$ is the local position specific diffusion coefficient, which can be calculated by the Hummer’s positional autocorrelation extension of Wolf-Roux estimator^57^. A detailed discussion on the computation of local diffusion coefficient $D\left( z \right)$ has been reviewed elsewhere^58–60^.

#### Area per lipid (APL)

Molecular packing of a lipid bilayer is described by APL. In molecular dynamics simulation, a pure lipid bilayer has the normal along z direction. The APL can be calculated by following equation:

$$ {\text{APL}} = { }\frac{{L_{x} L_{y} }}{{N_{lipid} }} $$

where,$$ L_{x} , L_{y}$$ is the box length in x, y direction. $$N_{lipid}$$ indicates the total number of lipid molecules in one leaflet.

#### Order parameter

The ordering of lipid acyl chains was determined by the calculation of order parameter S_CD_. This quantity can be directly compared to experimental S_CD_ values obtained by ^2^H NMR or ^1^H-^13^C NMR. Since S_CD_ is also a measure of relative orientation of the C-D bonds with respect to the bilayer normal, it can be calculated according to the following equation:

$$ S_{CD} = 0.5\left( {3cos^{2} \theta - 1} \right) $$

where $\theta$ is the angle between the bilayer normal and vector joining C-D (actually C-H in the current simulation), and <  > represents an ensemble average.

#### Statistical geometry

To investigate the effect of drug permeation on the structure of membrane, the statistical geometry approach was used. The analysis was initiated by partitioning the space contained in the simulation box by means of Delaunay Tessellation^61^. The detailed description of the method can be found elsewhere^50,60,61^. In this study, the Delaunay tetrahedron was formed between the heavy atoms of POPC (Phosphate), cholesterol (Oxygen) and water (Oxygen). The local structure of a system can be quantified by the distortion of Delaunay tetrahedra, which has been defined in terms of a parameter called tetrahedrality (T) defined as follows^62^:

$$ T = \sum \left( {\frac{{\left( {l_{i} - l_{j} } \right)^{2} }}{{15\overline{{l^{2} }} }}} \right) $$

where $l_{i} , l_{j, } \overline{{l^{2} }}$ are the edge length and the mean length of a tetrahedra, respectively. For a perfectly regular tetrahedron, the tetrahedrality is 0; an increasing deviation from regularity causes a corresponding increase in the parameter.

The free energy, diffusion and resistivity were calculated using umbrella sampling, while the other properties were derived by classical all-atom MDS data.

### Generation of anti-Withaferin-A and anti-Withanone antibodies

Wi-A and Wi-N were extracted from dried Ashwagandha leaves as described in a previous study^15^. Mixture of Wi-A/Wi-N (1:1) along with Freund’s adjuvant was used as antigen to immunize the mice for monoclonal antibody generation. The resulting clones (~ 30) were screened with affinity ELISA and one clone L7C3-6 (WiNA Ab) was isolated that reacted to Wi-A and Wi-N in fixed cells. The clone was established as hybridoma. Affinity purified antibody was used for this study.

### Cell culture and treatments

Human normal (TIG-3, MRC5 and WI38) and cancer (breast carcinoma-MCF-7, melanoma-G361 and osteosarcoma-U2OS) cells were obtained from Japanese Collection of Research Bioresources (JCRB, Japan). The cells were authenticated by the source. Cells were frozen in -80 °C and LN_2_ in multiple vials and were cultured in Dulbecco’s modified Eagle’s medium (DMEM; Gibco BRL, Grand Island, NY, USA) and treated either with Wi-A or Wi-N at about 60% confluency. Internalization of Wi-A and Wi-N was detected by immunostaining with the anti-WiNA Ab raised in our laboratory. The treated cells were also immuno-stained with a variety of other antibodies that included anti-γH2AX (Millipore #07-627), anti-ATR1 (Abcam, #ab4471), anti-CHK1 (Cell Signaling #2345S), anti-p53 (Santa Cruz. #sc-126) and anti-CARF antibody was raised in our laboratory^63^.

## Results and discussion

### Membrane architecture

In order to validate our simulation protocol, few parameters like Area Per Lipid (APL), electron density, Lipid order and Tetrahedrality were calculated in control as well as in presence of Wi-A and Wi-N molecules, which were compared to the experimentally observed values.

### Area per lipid (APL)

The compactness of a biomembrane is measured by area per lipid. Addition of a permeant alters the density of a membrane. For the liquid-crystalline phase of POPC membrane, a number of APL values have been reported, depending upon the temperature: 68 Å^2^ at 303K^64^, 62 Å^2^ at 323 K, 66 Å^2^ at 310K^65^, 63 Å^2^ at 310K^66^, 62 Å^2^ at 310K^67^. In our MD simulations, the APL for the control POPC membrane was ~ 60.22 ± 0.06 Å, which increased slightly to 60.27 ± 0.055 Å^2^ and 60.28 ± 0.06 Å^2^ in the presence of Wi-A and Wi-N respectively. This was in line with a previous report that used POPC membrane with similar simulation conditions^68^. This suggested that the passage of drug molecule has no overall major effect on the membrane architecture. The experimental APL values, as reported earlier, generally vary from 63.0 to 68.3 Å^2^^64–67^. The discrepancies between the calculated and the experimental values of APL could be attributed to the presence of cholesterol molecules in the biomembrane. Using ^13^C-NMR spectroscopy on POPC membrane, it has been earlier reported that addition of 50% cholesterol caused ordering of the POPC membranes by losing the entropy^69^.

### Electron density profile

**Figure 2:**
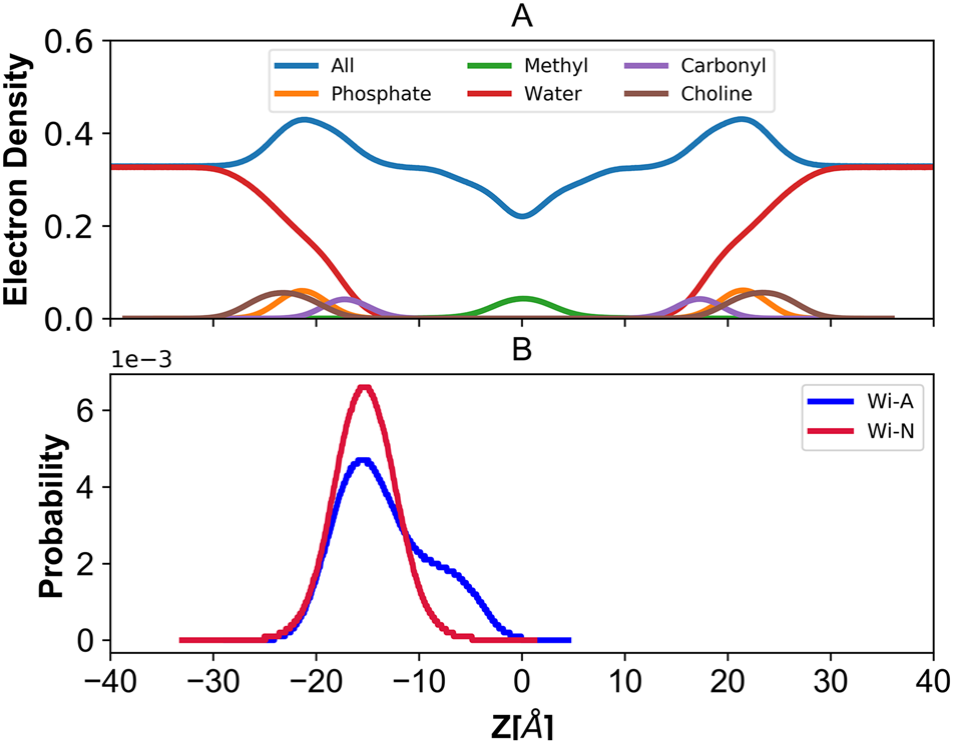
(**A**) Electron density profile of different components in Control POPC membrane. (**B**) Density profile of Withaferin-A (Wi-A) and Withanone (Wi-N) with respect to normal membrane.

Generally, thickness of the bilayer is determined by the electron density profile measured by the X-ray scattering of liquid-crystalline membranes^64,66,67^. The electron density profiles (EDP) were calculated by assuming an electron charge equal to the atomic number minus the atomic partial charge located at the center of each atom. The electron density profile decomposed into various groups as water, drug molecules, choline (CHOL), phosphate (PO_4_), glycerol, carbonyl (COO), methylene (CH_2_), unsaturated CH=CH and terminal methyl’s (CH_3_) is plotted in Fig. 2. The density profile for POPC bilayer in control is shown in Fig. 2A. Almost similar trends were obtained in case of Wi-A and Wi-N (Supplementary Fig. 1). From Fig. 2A, it is clear that in case of all atom density, three distinct domains are visible. While the flat region >|25| Å represents an aqueous phase, the unimodal distribution peak at ~ 15 Å corresponds to the interface region, containing lipid head-groups and water. Similar peaks were also observed for the phosphate atom, choline and carbonyl groups. The drop in the density represents the inner part of the bilayer, where the alkyl chains of the phospholipid reside. The decrease in water density, from bulk to middle of bilayer, indicates that no water molecule has traversed the lipid tail. In each case (Fig. 2A, Supplementary Fig. 1), the electron density profiles were symmetrical, almost overlapping, with water penetrating up to the carbonyl groups, leaving the terminal methyl groups dehydrated in agreement to the experimental results. The simulated electron density profile indicated that the parameters chosen for the MD simulations reproduces the membrane geometry at a good level. On the contrary, the electron density profile of Wi-A and Wi-N clearly showed that the preferable locations for withanolides (in due course of MD simulation) was close to lipid head group region (Fig. 2B).

### Order parameter (S_CD_)

**Figure 3:**
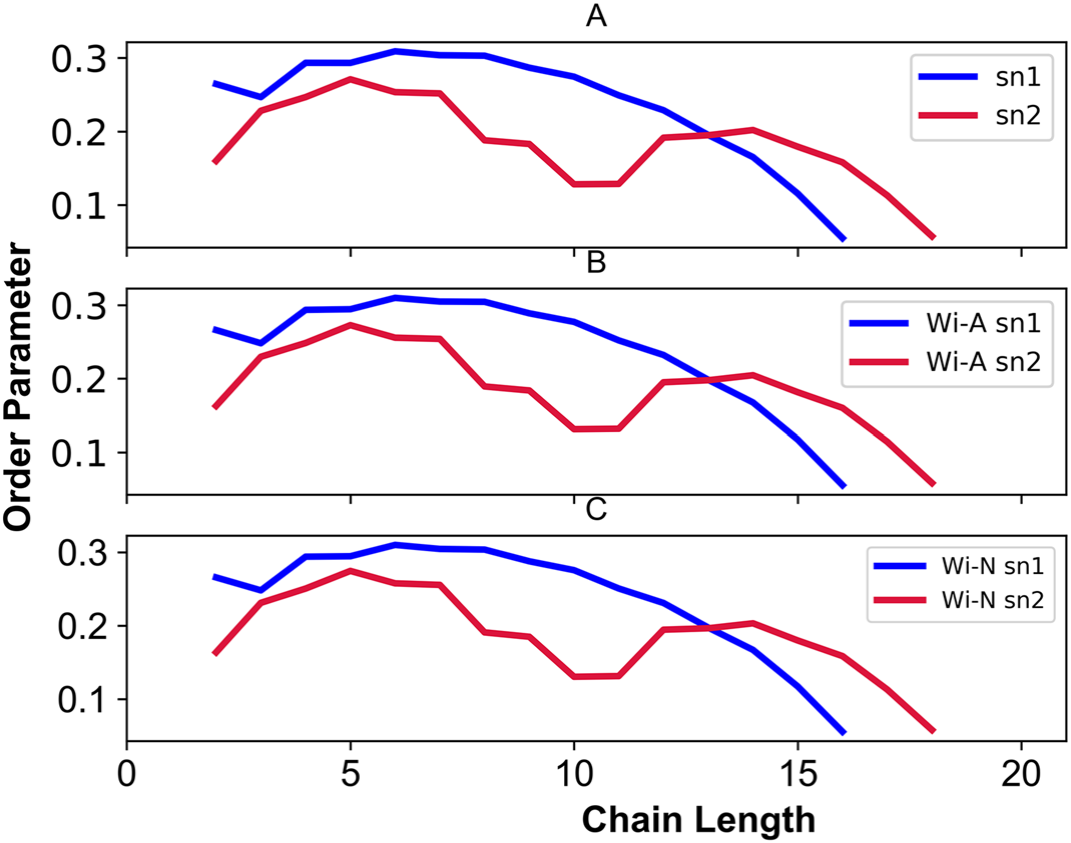
Order parameter (S_cd_) as function of the position of the carbon atoms. The panels (**A**–**C**) correspond to the S_cd_ of control, Withaferin-A (Wi-A) and Withanone (Wi-N), in sn-1(- -) and sn-2 (- -) hydrocarbon chains.

The order parameters of POPC membrane acyl chain in presence of Wi-A and Wi-N are shown in Fig. 3. The calculated order values follow a similar trend as previously reported in simulations and experiments^70,71^. The carbon-2 atoms of the sn-1 and sn-2 chains displayed different order parameters owing to different alignment of the acyl chains in the region. Experimentally, it has been observed that the S_CD_ of C-D bonds near the head group in the sn-1 chains are greater than the sn-2 chains^71^. Similar behavior was observed for the calculated lipid systems. The unsaturated chain of POPC showed a drop at the carbon 9 and 10 due to double bond. Further, the high S_CD_ value of sn-1 chain indicated a high order of acyl chain as compared to oleyl chain of POPC. The plot revealed that for the sn-1 chain (1) a maximum S_CD_ of 0.2 near the aqueous phase, (2) the S_CD_ is slightly high for the first five segments as a plateau and (3) from the sixth carbon, the value gradually decreases up to ~ 0.07. In case of sn-2 oleyl chain, (1) a maximum S_CD_ of ~ 0.19 near the aqueous phase, (2) a distinguishing drop at C2^′^ to 0.15 and (3) a characteristic minima ~ 0.02 indicates the double bond of the oleyl chain^70^. The calculated order parameter showed the acceptable agreement between the experimental and calculated values. However, the small differences could be attributed to the inherent error of theoretical methods applied for the derivation of S_CD_ values from NMR spectroscopy and simulations.

### Voronoi/delaunay tessellation and interspatial arrangement

**Figure 4:**
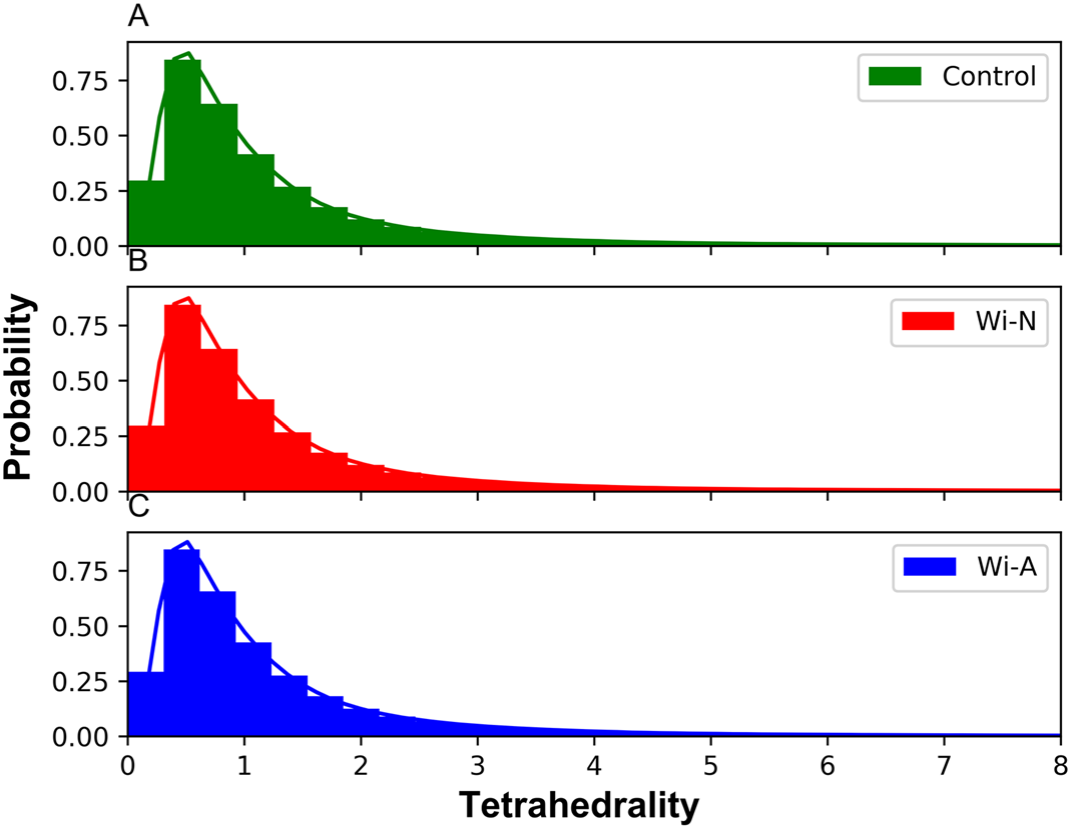
Distribution of tetrahedrality (T) values for control (**A**) Withanone (**B**) and Withaferin-A (**C**).

Traversing the membrane by a drug molecule is likely to result in the distortion of the spatial arrangement of the bilayer. Statistical geometry analysis provides an elegant approach to investigate the local geometrical arrangement of the molecules. Delaunay tessellation portions the 3D space into series of non-overlapping tetrahedra. In this study, the tetrahedrality distribution for control, Wi-N and Wi-A was uni-modal that peaked at ~ 0.5 depicting a regular arrangement of atoms, which was not perturbed by the traversing of bilayer by the drug molecules (Fig. 4). However, the long tail of tetrahedrality distribution, associated with low probability, indicated the formation of few irregular tetrahedral arrangements. Taken together, the variation of APL, electron density, Lipid order and Tetrahedrality variation suggested that the traversing of drug molecules had no effect on the over all architecture of the bilayer. Fraction of Native Contacts (Q) between drug and POPC lipid (Supplementary Fig. 4) showed that both the drug molecules had interaction preferences for the lipid tail region as compared to the polar head region. This behavior could be attributed to the hydrophobic nature of the drugs as well as lipid molecules. Further, Wi-A was observed to interact more with lipid tail region in comparison to Wi-N.

### Orientation and insertion depth of withanolides in the membrane

**Figure 5:**
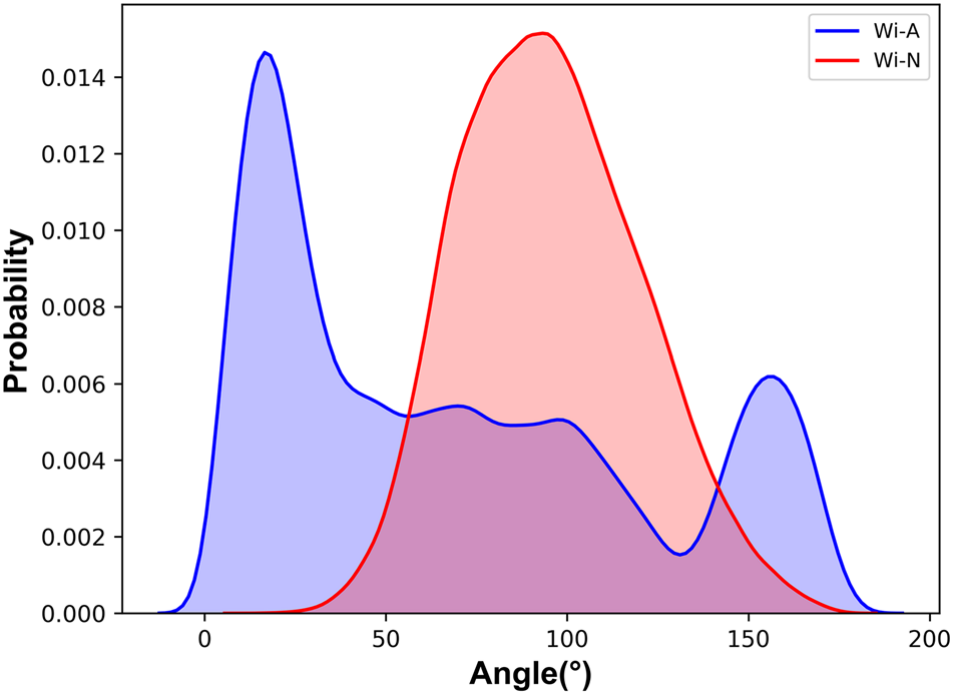
Tilting of withaferin-A (Wi-A) and withanone (Wi-N) molecules with respect to the membrane normal.

Withanolides are overall hydrophobic molecules (Fig. 1, Table 1). However, they carry different functional groups at various positions that influence their orientation and position of steroids in the membrane. The probability distribution for angle of Wi-A was calculated. It is defined by the atoms C18 “head” and C25 “tail” respectively that connected the two opposite positions with respect to bilayer normal. Similarly, for Wi-N, the vector was drawn between the C19 and C25 atoms. The Fig. 5 shows the tilt angle between membrane normal and steroidal axis, where both the drug molecules adapt different orientations. The multimodality of Wi-A tilt angle distribution suggested that it does not assume a single well-defined orientation, but instead has a wide distribution of orientation. The tilt angle distribution of Wi-N was unit-modal (that peaked at ~ 100°) that indicated a well-defined orientation. For instance, the conformation of Wi-A is imposed by the polar hydroxyl groups at the C13, C24, C27 atoms and methyl groups at C14, C16, C21, C27 position (Fig. 1). This particular configuration imposed a specific vertical orientation (i.e. parallel to bilayer normal) position, such that the hydrophobic groups were solvated by the lipid tails and the polar groups (O5 and O4) can form hydrogen bonds with other polar parts of the membrane^72^. In addition to orientation, another major degree of freedom was given by the positions of the withanolides relative to the membrane center of mass, which is the insertion depth in the membrane (Table 2). As expected, because of their overall lipophilic nature, these molecules tend to localize below or near the ester groups of POPC at the interface between polar or apolar regions of the membrane. Similar locations for other steroids are reported in literature^72^. The different orientations acquired by Wi-A and Wi-N while passing through the lipid membrane is shown in Supplemetary Figs. 2 and 3, respectively.

**Table 1:** The comparison of chemical and physical properties of the Withanolides.

Propeties	Withaferin-A	Withanone
Molecular weight	470.61 g/mol	470.61
SASA	726.72	706.52
Polar surface area	96.4	96.4
Hydrogen bond donor	2	2
Hydrogen bond acceptor	6	6
QlogKp	− 4.0	− 3.03
QlogS	− 5.14	− 5.4
logP	3.8	3.1
logD	3.58	2.2

*SASA* solvent accessible surface area, *QlogKp* predicted skin permeability, *QlogS* predicted aqueous solubility.

**Table 2:** Distance of the drug molecules from the phosphate atom of membrane (mean and standard deviation in 500 ns).

Withanolides	Membrane location
Wi-A	29.60 ± 8.12
Wi-N	31.16 ± 8.01

### Potential of mean force (PMF)

**Figure 6:**
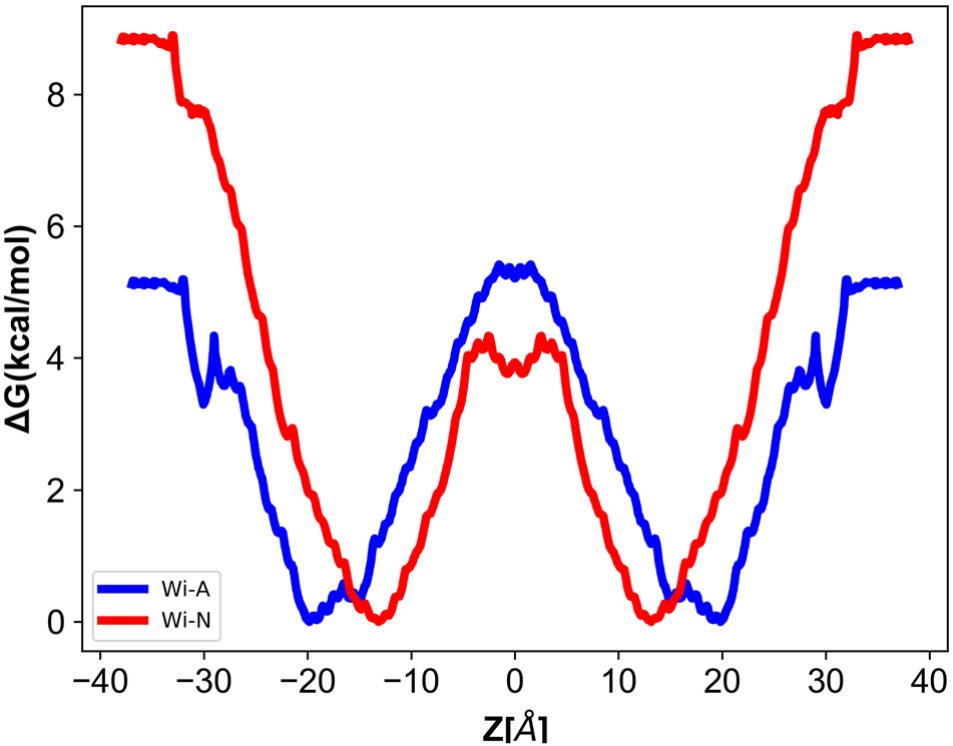
Change in the free energy (kcal/mol) of withaferin-A (Wi-A) and withanone (Wi-N) across the POPC-Cholesterol bilayer.

In the solubility-diffusion model, the calculation of PMF is a critical component for the estimation of membrane permeability. It corresponds to the relative solubility of a permeant in solution versus the membrane interior. The respective permeability of Wi-A and Wi-N from water phase to the interior of membrane is shown in Fig. 6. Considering the first part of the curves, from bulk water region to the polar head group, the high free energy values for Wi-N as compared to Wi-A is clearly evident. This indicated that the bilayer is a more preferable location for Wi-N than Wi-A. However, the portioning in bilayer was critical. While proceeding deeper in the bilayer that corresponds to the glycerol region, both the drug molecules displayed a free energy decrease, with a deep free energy minima at ~ 20 Å (Wi-A) and ~ 8 Å (Wi-N). Therefore, this region could be considered as the preferably partitioned region for both the withanolides. The rightmost part of Fig. 6 shows the change in free energy characterizing the permeants in hydrocarbon tail region (bilayer core). Here, both drugs showed an increase in free energy, highlighting the barrier effect inserted by the lipid hydrocarbon tail. However, the differences in free energy values were small for both the drug molecules. From the PMF, it is clear that the bulk region is unfavorable for Wi-N. The ΔG value between the bilayer midplane and for free energy minimum for Wi-N is also smaller than that for Wi-A. This implied a smaller barrier for passing from one leaflet to the other for Wi-N when compared to Wi-A.

The variation in free energy value for both permeants moving from bulk water phase into the membrane could be attributed to the increase in density (Fig. 2B). Density profile clearly showed that the preferable locations for withanolides were symmetric and close to lipid head groups (the densest region of bilayer). This location, as well as the tilt angle distribution (Fig. 5, Table 2) was in good agreement with the minima of the PMF implying that they were energetically favorable. For hydrophilic molecules, the lipid tail represented the main barrier to permeation, while for hydrophobic molecules, partition is more favored in the middle of a bilayer than at lipid/water interface. The difference in the PMF values can be attributed to the hydrophobicity of the drug molecules. logP is the measure of lipophilicity of a molecule in un-ionized water, while logD is the measure of the same in ionized environment (Table 1). As majority of known drugs are likely to be charged at physiological pH, using logP to describe their lipophilicity could be misleading. logP alone describes the partition coefficient of a *neutral* (uncharged) molecule, hence logP offers an advantage in such (*neutral* stage) cases. The difference in the value of logP and logD can also be used as a measurement for understanding the effect of charge distribution on the surface of molecule. Because POPC membrane is highly chemically diverse, its hydrophilic layers provided charged environment to the incoming molecules. The logP value of Wi-A (logP = 3.8) was slightly higher than that of Wi-N (logP = 3.1), but logD value of Wi-A (logD = 3.58) was much higher than that of Wi-N (logD = 2.2). Besides, the interior of lipid membrane favored the transport of lipophilic molecules. This suggested that in charged environment, Wi-A is more lipophilic than Wi-N and hence this charge distribution over the surface of Wi-A and Wi-N could explain the selective permeability of the Wi-N. Therefore, the free energy for passage of Wi-A molecules in the lipid head region is lower, while Wi-N experiences a high-energy barrier while crossing the membrane-water region. A video showing the permeation of Wi-A across the normal bilayer is included in the supplementary material (Supplementary Video 1: Wi-A_permeability.mov).

### Diffusion and resistivity

**Figure 7:**
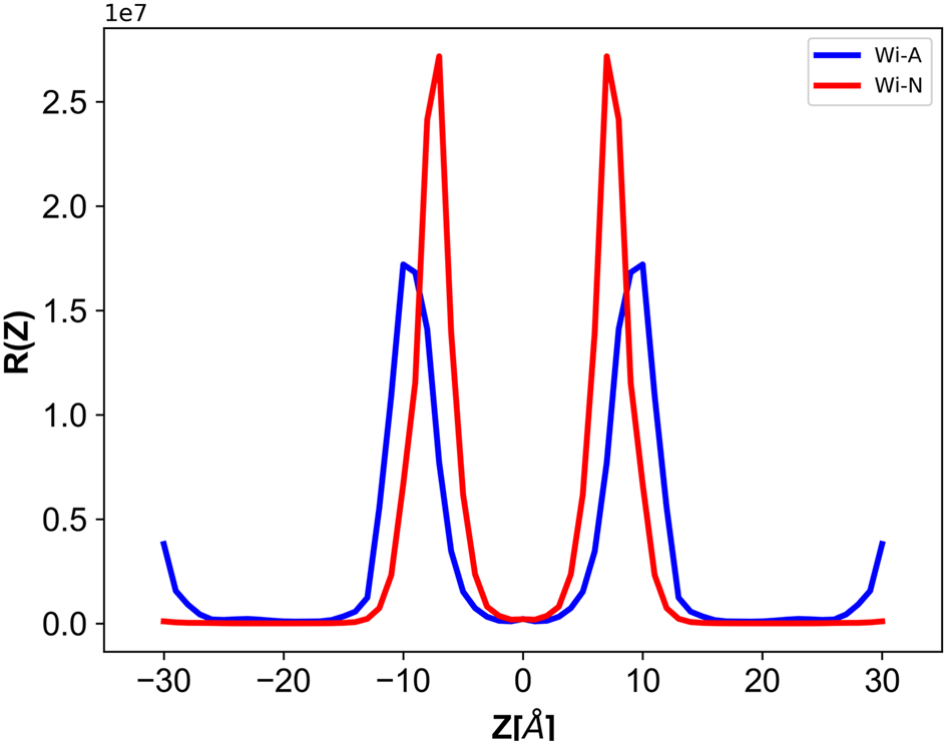
Diffusion and Resistivity profiles of withaferin-A (Wi-A) and withanone (Wi-N) molecules across the bilayer.

The second key component of the solubility-diffusion model is the diffusivity/Resistivity. The above results indicated that transfer of Wi-A across the bilayer tail region is less energy-driven and hence favorable as compared to Wi-N. Further, to check the resistivity exhibited by the bilayer on the drug molecules, the resistance exerted by the bilayer on both the natural drug molecules were calculated and shown in Fig. 7. The variation in resistivity showed a high resistance for Wi-N molecule in the lipid head region, while the resistance experienced in the bilayer tail region is almost similar for both Wi-A and Wi-N drug molecules. From the electron density (Fig. 2B), PMF (Fig. 6) and resistivity variation (Fig. 7), it is evident that the head part of the membrane was primarily resisting the passage of natural drug molecule across the bilayer, while the hydrophilic region was offering almost similar barrier/resistance for both the withanolides. Between Wi-A and Wi-N, the lipid head group region seemed to play a key role in determining the easier passage of Wi-A drug molecule as compared to Wi-N drug molecule.

### Solvation and hydrogen bond

**Figure 8:**
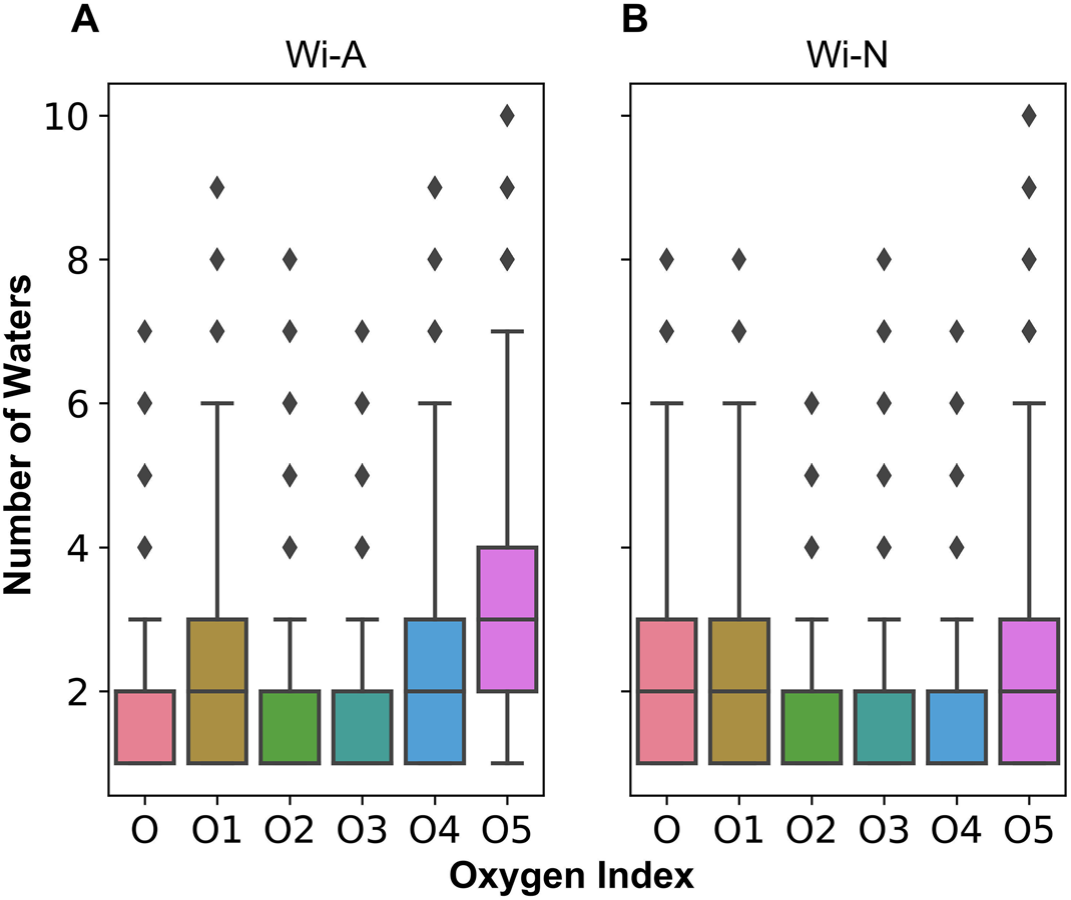
The average number of water molecules in the first hydration shell of oxygen atoms of Withaferin-A (**A**) and Withanone (**B**) molecules. The indexes of oxygen atoms are as mentioned in Fig. 1.

Though both the withanolides possess similar structure, the results indicated a favorable passage for Wi-A across the bilayer polar region. To understand the differences associated with this phenomenon, the average numbers of water molecules in the first hydration shell (number of water molecules in the 3.5 Å radius of oxygen atom) were calculated for the drug molecules. The boxplot representation showed a higher solvation of Wi-A as compared to Wi-N, where the average solvation of epoxy oxygen is similar for both the permeants (Fig. 8). Though subtle differences could be observed for other oxygen atoms, in Wi-N, the average water solvation was ~ 6 water molecules for rest of five oxygen atoms. However, for Wi-A, all the oxygen atoms were more solvated with ~ 7 water molecules. Further to get a clear picture of water dynamics around the drug molecules, the hydrogen bond lifetime between water molecule and drug oxygen atoms was investigated. Oxygen of water has a natural tendency to form hydrogen bond with other electronegative atom and hydrogen atom of water has the similar tendency to form hydrogen bond. Both the drug molecules have several –OH group that can form hydrogen bonds with water molecules; both water and drug molecules could act as hydrogen bond donor and acceptor in the formation of hydrogen bond. Depending upon the accessibility as well as the ambient environment, the interaction between the water and drug molecules may differ. Therefore, the calculation of hydrogen bond lifetime was expected to provide a measure for the elucidation of water dynamics around drug molecules leading to the favorable passage of Wi-A when compared to Wi-N. The maximum lifetime (in picoseconds) of hydrogen bond formed between water and the terminal O4 (~ 4.98) and O5 (~ 5.10) oxygen atom of the Wi-A as compared to other oxygen atoms of Wi-A is shown in Table 3. While, in case of Wi-N the average maximum lifetime was found to be ~ 3.27 ps and ~ 3.69 ps respectively for O4 and O5 atoms.

**Table 3:** Maximum hydrogen bond lifetime (in picosecond) between the oxygen atoms of Withaferin-A and Withanone drug molecules.

Oxygen index	Withaferin-A	Withanone
O	2.93	2.29
O1	3.58	2.62
O2	2.89	2.06
O3	1.32	1.11
O4	4.98	3.28
O5	5.10	3.70

Residue indexes for the oxygen atoms are as mentioned in Fig. 1

### Radial distribution function (RDF)

**Figure 9:**
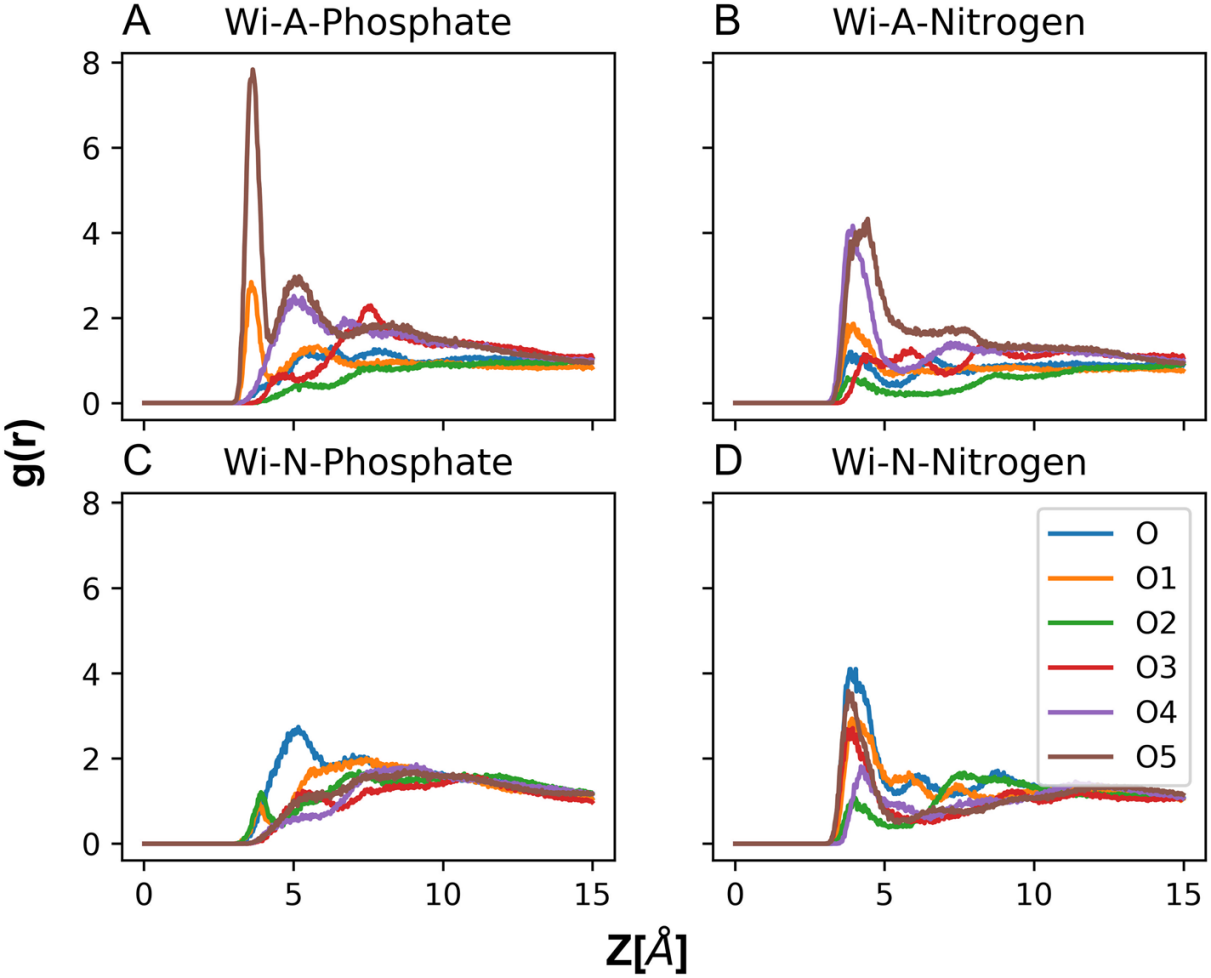
Radial distribution function (RDF) between oxygen atoms of Withaferin-A (Wi-A) and Withanone (Wi-N) with the lipid phosphate (**A**,**C**) and nitrogen (**B**,**D**) atoms. The index and color code for each oxygen is as mentioned in the top right of (**A**).

This analysis was carried out to track the passage of Wi-A molecule, which forms hydrogen bond with water, phosphate and oxygen atoms of the POPC atoms. The RDF between the oxygen atoms of Wi-A and lipid phosphate (Fig. 9A) and lipid nitrogen (Fig. 9B) was plotted. The nearest neighbor peak was clearly visible at ~ 3.5 Å and 4 Å for the phosphorus and nitrogen groups respectively. In case of phosphorus, the second nearest-neighbor peak was also pronounced at ~ 5 Å. Among all the oxygen atoms of Wi-A, a strong interaction with lipid phosphate was observed with O5 followed by O1 and O4 atom. Similarly, the O5 and O4 atoms again showed a high interaction with the lipid nitrogen atom. In case of Wi-N, the epoxy oxygen seemed to interaction with the lipid phosphorus and nitrogen atoms; however, the strength of this interaction was small as compared to Wi-A (Fig. 9C,D). Though both the molecules possess similar structure, Wi-A could still cross the bilayer proficiently but not Wi-N. The difference between two molecules actually lies in the distribution of single hydroxyl group. In Wi-A, the hydroxyl group is attached to the terminal lactone ring, whereas in Wi-N, hydroxyl group is attached to the middle of steroidal ring. Though the number of total polar atoms in Wi-A and Wi-N remains the same, the position of polar atoms makes a big difference in the charge distribution over their surface (Fig. 1, log D values). The terminal arrangement of polar atoms in Wi-A provided it an advantage over the distributed polar atoms in Wi-N. Grouping of the polar atoms at just the terminals made the middle surface of Wi-A continuously non-polar favoring interactions with both hydrophilic and hydrophobic environment. Grouped polar atoms are more capable to face the hydrophobic environment as their own interactions can reduce the total exposed surface area. From the high solvation (Fig. 8) of terminal polar groups (O4 and O5) in Wi-A as well as the strong hydrogen bond lifetime (Table 3) of same atoms along with the RDF analysis (Fig. 9), it can be interpreted that the interaction of terminal O5 and O4 atoms of Wi-A with polar atoms (water, lipid nitrogen, and lipid phosphate) of system enables the smooth transverse of Wi-A. While for Wi-N, no such favorable interactions were found; hence the passage of Wi-N across the normal membrane was associated with high-energy cost.

### Experimental evidence to the differential absorption of Wi-A and Wi-N by normal cells

**Figure 10:**
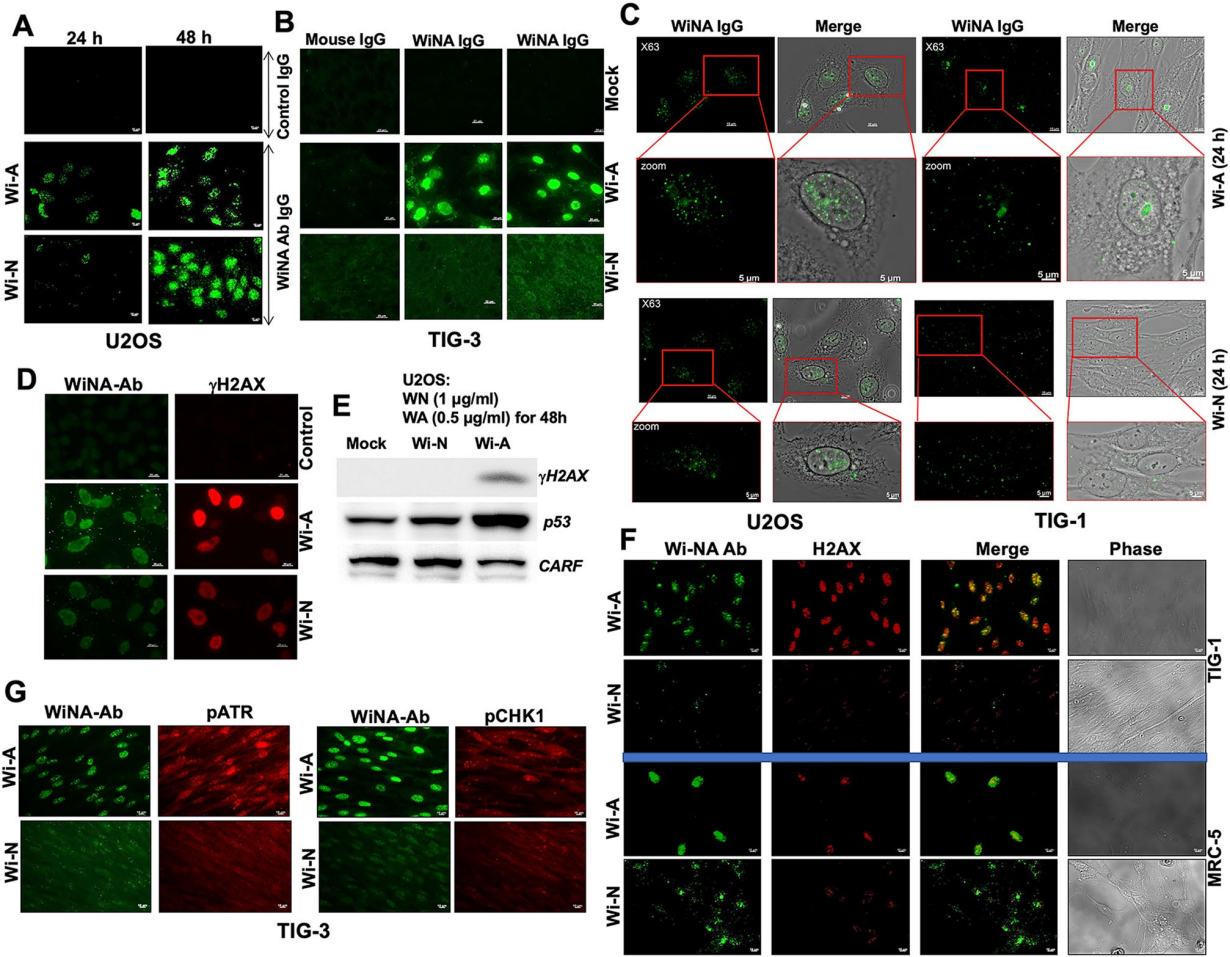
Experimental validation of differential permeation of Withaferin-A (Wi-A) and Withanone (Wi-N) through cell membrane. (**A**) Human cancer cells (U2OS) treated with Wi-A/Wi-N were immunostained with anti-WiNA antibody showed their presence in cells and predominantly in the nucleus. Cells treated for 24 h showed Wi-A, but not Wi-N. Cells treated for 48 h showed both Wi-A and Wi-N. (**B**) Normal cells treated with Wi-A for 48 h showed high intensity of Wi-A staining in the nucleus; Wi-N treated cells showed faint cytoplasmic staining. (**C**) High resolution images showing the presence of Wi-A predominantly in the nucleus of U2OS and TIG-3 cells. Wi-N showed weak nuclear staining U2OS cells, and pan-cytoplasmic staining in normal cells. (**D**) Wi-A/Wi-N treated cells were examined for DNA damage marker protein γH2AX showed its higher upregulation and activation in Wi-A, as compared to Wi-N treated U2OS cells. (**E**) Upregulation of γH2AX, p53 and downregulation of CARF in Wi-A, but not Wi-N, treated cells. (**F**,**G**) Wi-A, but not Wi-N, treated normal (TIG-3 and MRC5) cells showed increase in γH2AX, pATR and pCHK1.

In order to provide the experimental evidence to the above membrane permeability analysis, we generated, for the first time, antibodies to Wi-A and Wi-N. As shown in Supplementary Fig. 5, 3/96 clones that initially showed reactivity to Wi-A and Wi-N were developed into hybridoma and were examined for their reactivity to Wi-A and/or Wi-N. We found that the one clone (L7C3-6) was able to detect Wi-A and Wi-N in cells treated with either purified compounds or i-Extract containing Wi-A and Wi-N as major components. The clone was designated as WiNA antibody. We next used anti-WiNA antibody to detect Wi-A/Wi-N in human cancer cells treated with purified Wi-A or Wi-N; isotype matched secondary antibodies were used as negative control. As shown in Fig. 10A, higher level of both Wi-A and Wi-N were detected in cells treated for 48 h as compared to 24 h. We extended the analysis to human normal cells. Wi-A, but not Wi-N, was detected in the normal cells incubated with the purified compounds for 48 h (Fig. 10B). Furthermore, we found that Wi-A and Wi-N staining in the nucleus, which was confirmed by confocal microscopy (Fig. 10C). Wi-A was detected clearly in the nucleus of cancer as well as in normal cells. In contrast, Wi-N was detected in the nucleus of cancer cells only; normal cells showed weak pancytoplasmic staining (Fig. 10C). In order to further support these findings, we examined the expression of several proteins that have been shown to be involved in anticancer activity of Wi-A and Wi-N. As expected, Wi-A caused stronger up-regulation of γH2AX and p53, markers of DNA damage response in cancer cells (shown in Fig. 10D,E). Furthermore, only the cells treated with Wi-A showed decrease in CARF protein signifying apoptosis (Fig. 10E). The same was not observed in Wi-N treated cells. In light of these findings that endorsed reliable detection of Wi-A/Wi-N in cells with our antibodies and in order to further validate the computational predictions on their differential permeability, we expanded the analysis in human normal cells. We found that Wi-A showed strong nuclear staining both in TIG-1 and MRC5 cells and associated with activated DNA damage response as determined by increase in γH2AX foci, nuclear pATR and pCHK1 (Fig. 10F,G). In contrast, Wi-N showed faint pancytoplasmic staining. Of note, increase in the DNA-damage marker proteins were not detected in normal cells. These data confirmed the MD predictions for the differential permeability of Wi-A and Wi-N through cell membrane and suggested a mechanism for their differential activity, at least, in part.

In this study, a detailed computational analysis characterizing the permeation of Wi-A and Wi-N, two natural drug molecules across the lipid bilayer was conducted. We report that the permeation of Wi-A and Wi-N does not affect the membrane integrity. The values obtained by area per lipid, electron density and order parameter, and tetrahedrailty were almost similar for bilayer in control and as well as in presence of drug molecules, suggesting no change in the overall architecture of the bilayer. Next, the PMF profile showed that the permeation of Wi-A across the bilayer was associated with low energy barrier and therefore, could be considered as a favorable transverse for Wi-A as compared to Wi-N. While the lipid head group region appeared as a determining region for the movement of drugs across the bilayer, the variation of resistivity profile confirmed the same. The polar head group region appeared to exert a high resistance on the passage of Wi-N as compared to Wi-A. Further, to elucidate the mechanics for this behavior, the solvation, RDF and hydrogen bond dynamics were calculated, which clearly indicated that the high solvation of terminal hydroxyl group (O5) along with tight interaction with polar head group atoms (phosphate/nitrogen) of the membrane, facilitate a smooth passage for Wi-A. The logP and logD values associated with the Wi-A and Wi-N supported these results. In spite of the fact that Wi-N also has similar structure like Wi-A, the lipophilicity of the molecule was less as compared to Wi-A that may account for its weak permeation through the membrane. This study revealed that the subtle difference associated with the positioning of functional groups can play a major role in transportation of drug molecules across the bilayer. The experimental data using unique Wi-A and Wi-N antibodies demonstrated that Wi-A indeed proficiently entered the cells and was detected in the nucleus by immunostaining. All together the calculated free energy profile for the permeation of Wi-A and Wi-N were qualitatively matching with the experimental observation. Further studies enrolling varying drug concentrations and more membrane models including (varying composition and density, representing different normal and cancer cell types and intracellular structures) are warranted. The study is an important step towards understanding the molecular basis of permeability of natural drug molecules.

## Supplementary Information

Supplementary Information
