# Supplementary material for: Molecular dynamics simulations and experimental studies reveal differential permeability of withaferin-A and withanone across the model cell membrane
Source: Sci Rep. 2021 Jan 27;11:2352. doi: 10.1038/s41598-021-81729-z (PMC7840742; doi:10.1038/s41598-021-81729-z)
Supplement: Supplementary file 1 — Supplementary Information [file 41598_2021_81729_MOESM1_ESM.pdf]

Molecular dynamics simulations and experimental studies reveal differential permeability of withaferin-A and withanone across the model cell membrane

Renu Wadhwa[1,#], Neetu Singh Yadav[2,#], Shashank P Katiyar[2], Tomoko Yaguchi[1], Chohee Lee[1,3], Hyomin Ahn[1,3], Chae-Ok Yun[3], Sunil C Kaul[1,*] and Durai Sundar[2,*]

Supplementary Information

Supplementary Methods

Parameterization of the Withanolides: The geometry optimization and the charge of Wi-A and Wi-N molecules were calculated at 6-31G* level by using GAMESS-US [1] and further refinement was carried out by using Restrained Electrostatic Potential Fit (RESP) using the RED-Tool package [2].

The equilibration protocol: The solvated bilayer in the simulation box was equilibrated using a slightly modified version of the multi-step protocol of Chachra and Rizzo [3]. Briefly, the procedure consisted of alternating cycles of steepest descent (500 steps) and conjugate gradient energy minimization (500 steps) followed by position restrained MD with strong harmonic restraints on all non-hydrogen atoms of the lipid and solvent. In successive steps, the positional restraints were gradually relaxed first on the solvent atoms and later on the lipid atoms ultimately with 500 ps of unrestrained MD simulations. All energy minimization and MD simulations were run using the SANDER modules of the AMBER18 program suite. Force-field parameters for the drugs and water molecules were taken from the lipid14 parameter-set [4] built within the AMBER program suite [5].

References

1. Schmidt MW, Baldridge KK, Boatz JA, et al (1993) General atomic and molecular electronic structure system. J Comput Chem 14:1347–1363. https://doi.org/10.1002/jcc.540141112

2. Dupradeau F, Pigache A, Zaffran T, et al (2010) The RED. tools: advances in RESP and ESP charge derivation and force field library building. Phys Chem Chem Phys 12:7821–7839. https://doi.org/10.1039/C0CP00111B
3. Chachra R, Rizzo RC (2008) Origins of Resistance Conferred by the R292K Neuraminidase Mutation via Molecular Dynamics and Free Energy Calculations. J Chem Theory Comput 4:1526–1540. https://doi.org/10.1021/ct800068v
4. Dickson CJ, Madej BD, Skjevik \AAge A., et al (2014) Lipid14: the amber lipid force field. J Chem Theory Comput 10:865–879
5. Case DA, Cheatham TE, Darden T, et al (2005) The Amber biomolecular simulation programs. J Comput Chem 26:1668–1688

Supplementary Video 1

Supplementary Video 1: A movie depicting the passage of Withaferin-A (Wi-A) molecule (pink color) across the lipid bilayer (cyan). For clarity, the waters and ions are removed.
https://owncloud.iitd.ac.in/nextcloud/index.php/s/GHxQgDQqsNwA99J

Supplementary Figures

Supplementary Fig. 1. **Electron density of control POPC (A) and POPC membrane in presence of Withaferin-A (B) and Withanone (C) molecule**. The profiles of different membrane components are plotted where the black color corresponds to the total electron density variation. Water density is shown in cyan. The lipid atoms are in different colors – phosphate (red), carbonyl (green), choline (dark blue), double bond (magenta), methyl (pink) and methane (light blue).

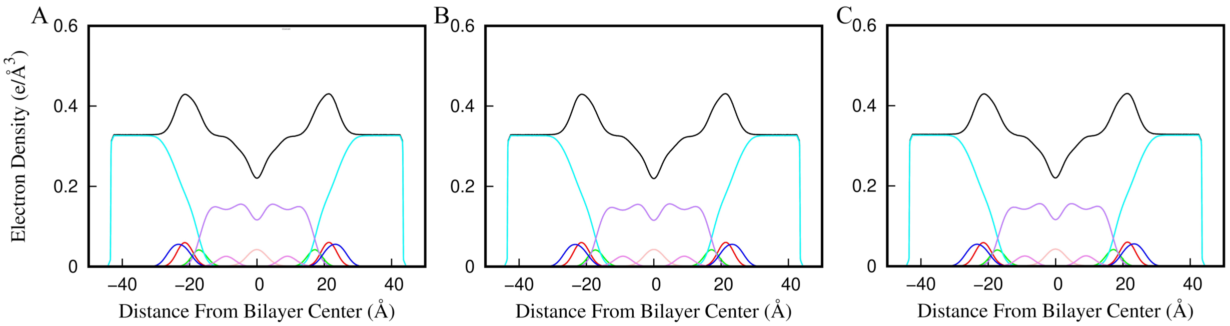

Supplementary Fig. 2. Permeation of Withaferin-A (Wi-A) through a lipid membrane. Wi-A crosses the lipid head group region, then diffuses rather slowly in the membrane interior. Wi-A is shown in yellow, the POPC lipids in tan, and cholesterol in light pink. The simulation time is indicated with each snapshot.

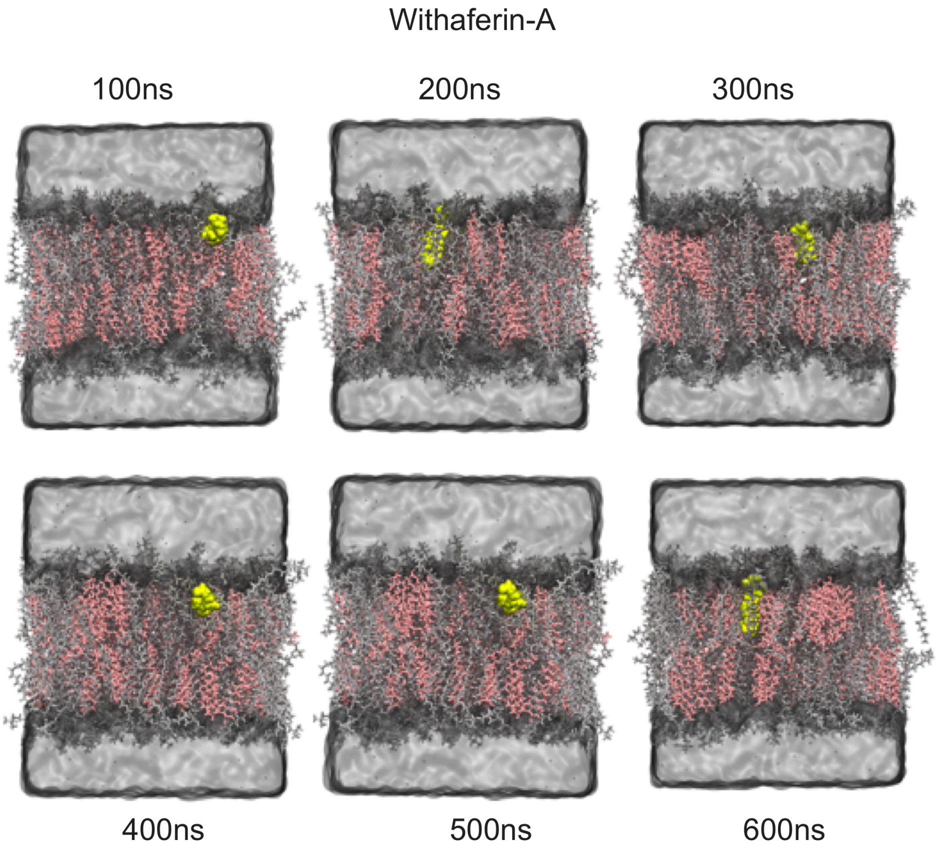

Supplementary Fig. 3. Permeation of Withanone (Wi-N) through a lipid membrane. After crossing the lipid head group region, Wi-N slowly diffuses in the membrane interior. Wi-N, the POPC lipids and cholesterol are shown in yellow, tan and light pink, respectively. The simulation time is indicated with each snapshot.

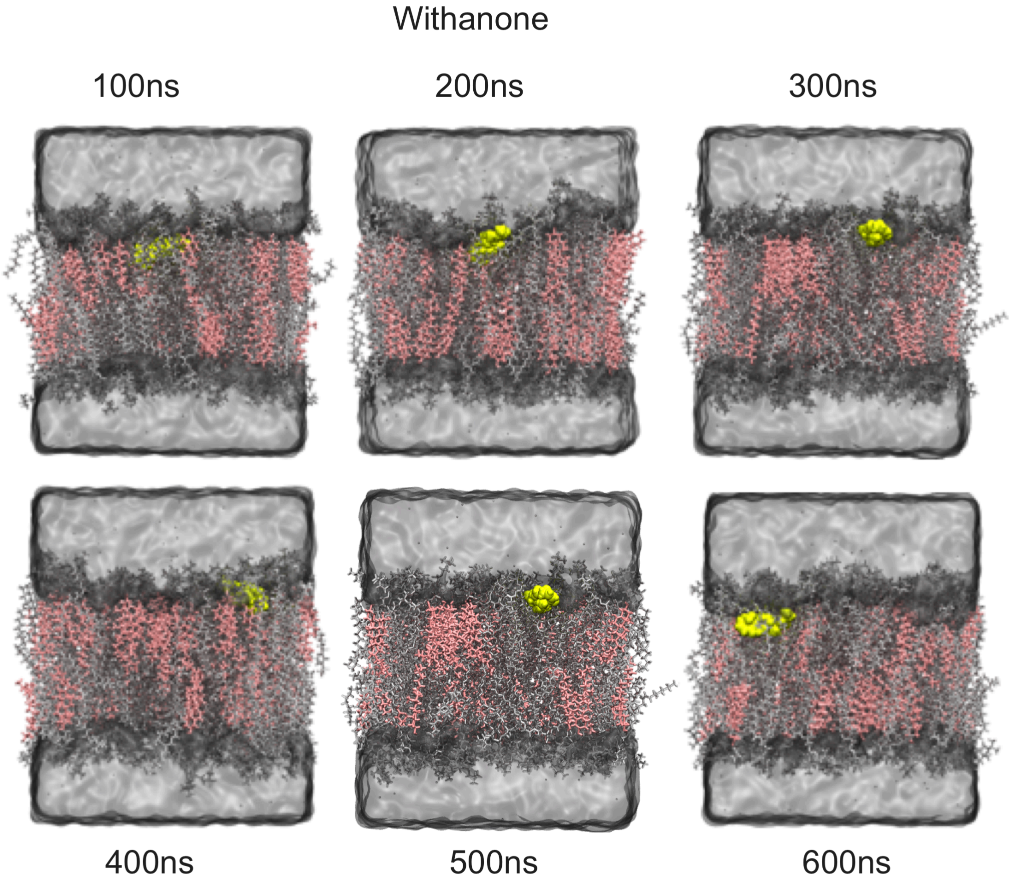

Supplementary Fig. 4. Fraction of Native Contacts (Q) between the drugs and the POPC lipid's head and tail. Wi-A molecule was making more interactions as compared to Wi-N.

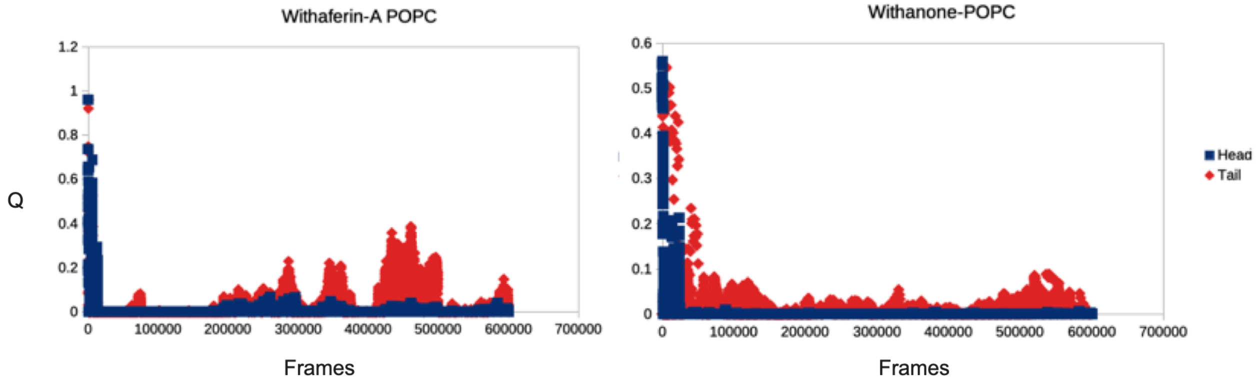

Supplementary Fig. 5. Detection of Wi-A and Wi-N in cells by specific antibodies. (A) Cells treated with Wi-A and Wi-N were immunostained with three clones (L2-D11-4, L2-D11-16 and L2-C3-6). Whereas, L2-D11-16 did not react either to Wi-A or Wi-N, L2-D11-4 detected Wi-A, and L2-C3-6 reacted both to Wi-A and Wi-N. (B) L2-D11-4 and L2-C3-6 were developed into hybridoma. Whereas affinity purified L2-D11-4 IgG did not detect either Wi-A or Wi-N, L2-C3-6 showed good reactivity to both.

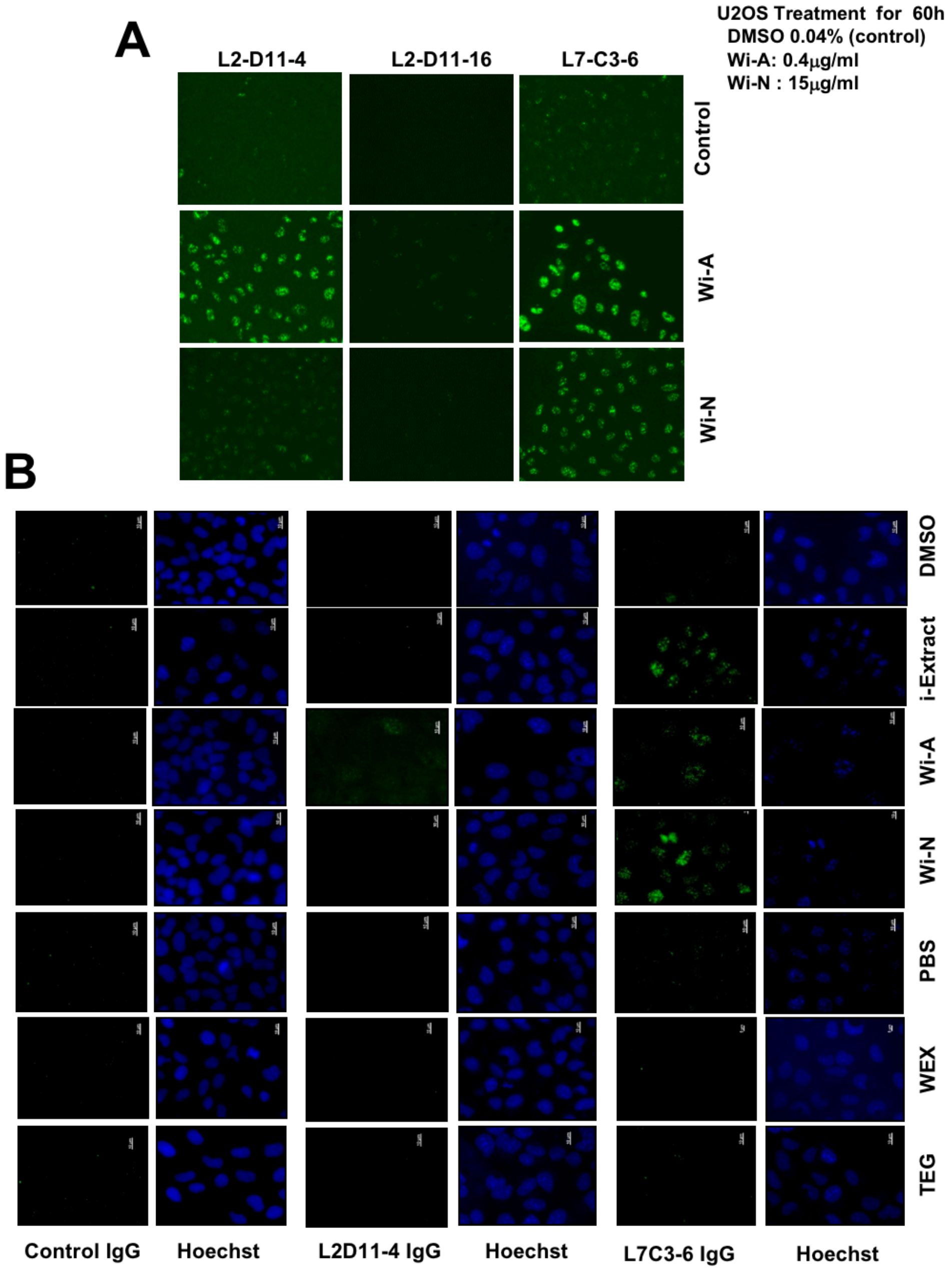

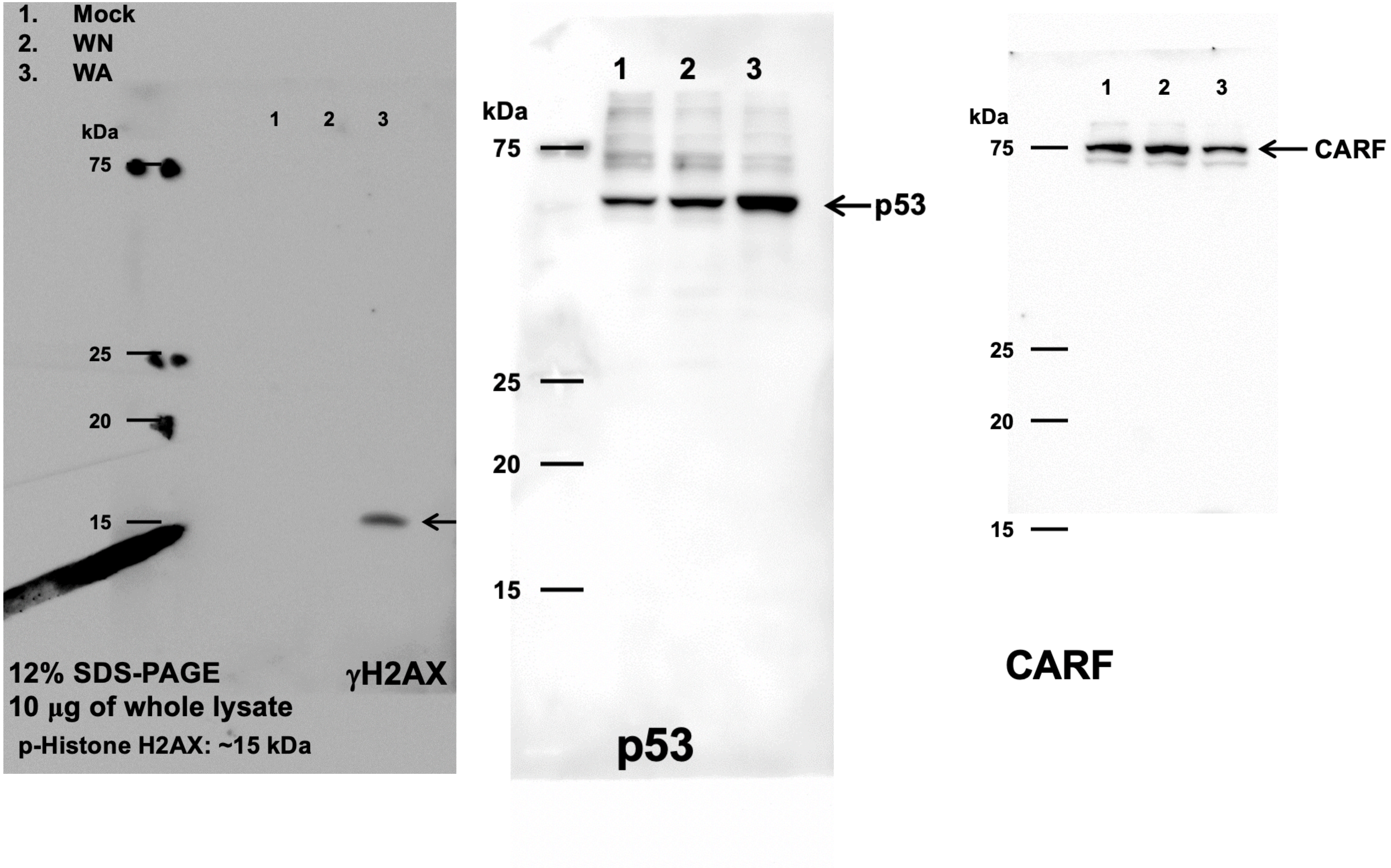

Western (FULL) blots to identify gH2AX, p53 and CARF proteins of interest presented in Figure 10 E
